# AI Experience Predicts Identification with Humankind

**DOI:** 10.3390/bs13020089

**Published:** 2023-01-21

**Authors:** Congyu Wang, Kaiping Peng

**Affiliations:** 1 College of Investigation, People’s Public Security University of China, Beijing 100038, China; 2 School of Social Sciences, Tsinghua University, Beijing 100084, China

**Keywords:** human identity, identification with humankind, AI experience, AI exposure, AI awareness, social identity theory

## Abstract

Artificial intelligence is becoming a potential outgroup of humans, which, according to social identity theory, may make humanity more salient. To explore how identification with humankind correlates to being exposed to artificial intelligence, we developed an AI Experience Questionnaire to measure this relationship and demonstrated that AI experience positively predicted human identity (Study 1a, *N* = 806). This correlation held when controlling for AI threats, educational level, international mobility experience, gender, and age (Study 2, *N* = 981, *M*_age_ = 27.55 ± 6.74; 448 males, 533 females). Study 1a also demonstrated that AI awareness—consisting of perceived anthropomorphism and perceived proximity—mediated the relationship between AI experience and human identity. This mediation model was replicated half a year later (Study 1b, *N* = 886). Moreover, a moderation analysis demonstrated that for both Easterners and Westerners, the correlation between AI experience and human identity was significantly positive; however, Western culture amplified the correlation (Study 3; *N* = 177, *M*_age_ = 32.35 ± 10.99; 90 Easterners, 87 Westerners). To conclude, persons with more AI experience may be more inclined to perceive AI as an outgroup of humans, and therefore AI experience positively predicts identification with humankind.

## 1. Introduction

Being exposed to AI may positively predict identification with humankind, because artificial intelligence, as a new category of entity [1], is becoming an outgroup of humans. The mere presence of an outgroup enhances ingroup identification according to social identity theory [2]. However, evidence of this effect has only been found within human social groups, such as national identity, racial identity, and organizational identification [3,4]. To what extent does this conclusion apply to the intergroup relations between humans and artificial intelligence? Research on AI-enhanced human identity occurs in the context of the threat of AI replacing human jobs and so does not only examine the impact of using AI [5,6]. The present study aimed to investigate the correlation between exposure to artificial intelligence and identification with humankind.

### 1.1. AI Exposure and AI Experience

Mere exposure to an outgroup is likely to strengthen ingroup identification in terms of human intergroup relations [2]. However, in some cases, non-human entities are seen as exacerbating human disunity. For example, many people worried that the COVID-19 epidemic would bring about a decline in globalization [7,8]. The immediate cause was delays and disruptions to global supply chains, but the underlying reason was the growing resource scarcity—exacerbating conflict and division. Another dramatic illustration is provided by some science fiction works, wherein humans are not a united group when confronted with aliens, but are separated into new categories (e.g., the Adventists in *The Three-Body Problem*, a science fiction novel written by Cixin Liu). However, unlike viruses or aliens, artificial intelligence is making more resources available in many areas through the development of machine learning, deep learning algorithms, computer vision, pattern recognition, natural language processing, and other technologies and applications [9]. People use AI with the clear understanding that it will help alleviate resource scarcity. Thus, as far as resource scarcity is concerned, AI may not exacerbate competition and conflict between human social groups. 

Despite the risk of resource scarcity, mere exposure to AI may not lead to stronger identification with humankind. This is because mere exposure is insufficient for users to gain the cognitive readiness to view AI as an outgroup of humans. In fact, it may not be AI exposure, but AI experience, that drives the perception of AI as a new category of entity. “AI experience” is the knowledge of how artificial intelligence works, gained through “AI exposure”. AI exposure reflects a situation in which a person is exposed to artificial intelligence, rather than the experience of using a specific application or technology, or the context in which threats from artificial intelligence are perceived. AI exposure can be seen as the sum of all AI-related events. These events include the number of hours spent using artificial intelligence technology and applications at home, in the office, and at school, as well as the hardware and software used. However, AI experience is defined as hands-on experience with artificial intelligence technology and applications that necessitates some understanding, through which knowledge of the fundamental principles that govern the functioning of AI can be gained. Thus, we inferred that persons with more AI experience would be more cognitively ready to see artificial intelligence as an outgroup.

### 1.2. Human Identity

We define human identity as identification with humankind, that is, seeing oneself as a member of the entire human race. People with a strong identification with humankind are concerned about the common well-being of the human community and are willing to help members of it [10]. For example, rescue workers who helped Jews escape the Holocaust all stated, “I am a member of the human family” [11]. This demonstrated that human identity eliminates discrimination between nations, ethnicities, and races. However, the significance of human identity to human communities is not only that it improves group relations, but also that it is related to individual characteristics and behaviors. Specifically, human identity is significantly positively associated with concern for global poverty and injustice, commitment to human rights, support for international charities, belief in justice, dispositional empathy, etc., whereas human identity is significantly negatively correlated to ethnocentrism, right-wing authoritarianism, social dominance, and other negative characteristics [10,12].

Because human identity has practical value both at the individual level and at the group level, considerable empirical research has been conducted on this topic. The first direct assessment of human identity perhaps dates back to the 1950s—the World-Mindedness Scale (WS), developed by Sampson and Smith in 1957 [13]. We adopted the Identification With All Humanity scale (IWAH) to measure human identity, because it is widely used to measure human identity and has demonstrated good levels of reliability and validity in various countries [10,11]. The IWAH has nine items, rated on a five-point Likert scale. A representative question is “How much do you identify with (that is, feel a part of, feel love toward, have concern for) each of the following?”, referring to three items (your community, your country, and “all humans everywhere”) that the respondent must score. To examine the unique association with human identity and to control for other identities, the raw score of “all humans everywhere” was subtracted from the score of “Chinese” to obtain a partial score for the final score.

### 1.3. The Relationship between Artificial Intelligence and Human Identity

Regarding the impact of artificial intelligence on human identity, previous research has demonstrated that increases in automation levels reduce discrimination and prejudice toward members of the outgroup, as higher automation levels bring the threat of replacing humans in their jobs [5,6]. These threats from artificial intelligence make human identity more salient, enhance panhumanism, and reduce intergroup bias in humans. However, humans are inherently sensitive to category cues from their early development stages [14], so the impact of artificial intelligence on human identity is not only related to the threats it poses. Similarly to the case of human social groups, the mere presence of an outgroup can make human identity salient [2]. Sharing the self-categorization as humankind, members from different countries or ethnicities are turning from “they” to “we”. In a word, AI experience enables people to be more inclined to perceive AI as an outgroup of humans, and thus it is probably positively correlated with human identity.

So far, few empirical studies have explored the psychosocial effects of AI experience, and few corresponding instruments for its measurement have been developed. However, this study could refer to many studies on computer experience. First, we drew on the content and structure of various computer experience questionnaires to develop an AI experience questionnaire [15,16,17,18]. Then, we aimed to test the hypotheses presented below through correlation analysis.

**Hypothesis** **1 (H1).** *AI experience positively predicts human identity.*

### 1.4. AI Awareness as a Mediator

Some point out that current AI cannot be considered truly intelligent because it performs inferiorly to humans in many areas [19]. However, this does not stop AI from affecting human identity, because the real impact on human identity comes not from artificial intelligence technology or applications, but the subjective construal of artificial intelligence as a social category. The principle of construal is one of the fundamental principles of social psychology. Social psychology has long emphasized the subjective construal of stimuli, in contrast to behaviorism, which only focuses on the relationship between stimuli and responses without examining the “black box” of the human brain. Human–computer interaction is primarily an issue of social psychology [20,21]. The Computers Are Social Actors (CASA) paradigm claims that the research methods used in social psychology could be used in the field of human–computer interaction [22]. Consequently, HCI research employs the user’s perception of the machine as a mediating variable (e.g., perceived anthropomorphism and perceived intelligence) rather than merely investigating the impact of objective technologies and applications on users [23,24]. This study was both a social psychology study and a human–computer interaction study, and we also used subjective perception as a mediator between AI experience and human identity. Specifically, we propose “AI awareness”, defined as the perception that artificial intelligence is comparable to humans in task performance. We argue that AI awareness is a direct predictor of the psychosocial effects of AI; AI awareness is influenced by—but not identical to—AI experience.

Not all categories of nonhumans are outgroups of humans. Being an outgroup necessitates two features: similarity and proximity. In other words, only two similar and proximate categories can be socially compared [25]. The social comparison between two categories is a necessary process for the social categorization that makes ingroup identity salient. For example, the California Golden Bears football team, representing UC Berkeley, is frequently compared to the Stanford Cardinal football team, but not the Stanford Cardinal baseball team, because they are not similar; nor is it compared to another college football team from a different division, because they would not be proximate. In summary, two features are essential prerequisites for artificial intelligence to be an outgroup of humans: (1) perceived anthropomorphism—the degree of similarity to humans attributed to robots; and (2) perceived proximity—a subjective perception of when artificial intelligence attains a human level. Thus, we selected perceived anthropomorphism and perceived proximity to operationalize AI awareness. We proposed that persons with more AI experience would be more likely to perceive it as similar and proximate to humans, and thus as an outgroup for the human race:

**Hypothesis** **2 (H2).** *AI awareness, consisting of perceived anthropomorphism and perceived proximity, mediates the relationship between AI experience and human identity.*

However, why is perceptual intelligence not one of the indicators of AI awareness? Although perceived intelligence is a common perceptual dimension associated with AI in previous studies, it does not compare to human intelligence [22]. The present study aimed to explore the impact of AI as a social category rather than the impact of AI as a tool. Both perceived intelligence and perceived proximity use the human level as a reference, while perceptual intelligence lacks this key reference point. Therefore, perceived intelligence was excluded from the indicators of AI awareness.

### 1.5. The Role of Culture

Cultural psychologists have found a wide range of differences between Eastern and Western cultures, influencing all aspects of people’s perceptions and behaviors [26]. How do culture and AI experience interact, if at all, in their effects on human identity? There are two competing propositions, which we labeled, respectively, the Eastern-amplified hypothesis and the category-based hypothesis.

The “Eastern-amplified hypothesis”, suggested that the correlation between AI experience and human identity is amplified in Eastern culture. It is likely that Easterners have more experience using artificial intelligence than Westerners. Because Westerners score highly on individualism and place emphasis on individual choice, personal freedom, and self-actualization [27,28], some researchers have pointed out that Westerners are more concerned about AI privacy risks and tend to perceive themselves as being exploited [29], which may increase their uncertainty about using artificial intelligence technology and applications [30,31]. Meanwhile, Eastern culture encourages an interdependent self-construal, and persons with a strong interdependent self-construal tend to feel more connected with others [32]. They may also be more willing to connect to artificial intelligence in person. This might be the reason why Easterners prefer to frequently interact with embodied intelligence, such as humanoid or animal-type robots, while Westerners prefer to interact with non-embodied intelligence, such as virtual assistants [33]. There is no empirical evidence confirming that Easterners use artificial intelligence more frequently than Westerners, but it is reasonable to infer that Easterners may have more AI experience and thus a better chance of perceiving AI as a human outgroup. Because Easterners have greater privacy risk perceptions and fewer face-to-face contacts with AI, the present study tested whether the association between AI experience and human identity is stronger in Eastern cultures.

The category-based hypothesis suggested that the correlation between AI experience and human identity is amplified in Western culture. Because of culturally distinct cognitive representations of social ingroups, processes consistent with social identity theory and self-categorization theory are most applicable to Westerners [34]. Specifically, in Western cultures, identification with ingroups tends to place a greater emphasis on categorization and intergroup comparison. In contrast, in Eastern cultures, ingroup identifications are not affected by categorical factors but solely determined by relational factors—maintaining harmony within groups, being sensitive to the needs and feelings of others, and being aware of the relationship structure within the group [35].

Nonetheless, empirical studies conducted within the context of Western culture revealed that AI use in the workplace significantly increases panhumanism and decreases outgroup discrimination in humans [5,6], which suggests that AI has the potential to enhance human identity for Westerners. Therefore, we tested both of these hypotheses by using cross-cultural research to determine in which cultures the AI experience is more strongly associated with human identity.

### 1.6. Current Research

Across four studies, we aimed to extend social identity research by analyzing whether artificial intelligence experience predicts identification with humankind. This task was essential for the following reasons: To begin with, the answers to these research questions could provide insights into the social psychology of human–machine interaction, especially the intergroup relations between humans and artificial intelligence. Secondly, by drawing on classic theories of intergroup relations in the human domain, we aimed to not only expand the theory and research on social identification processes but also demonstrate that artificial intelligence can serve as an outgroup and encourage people to consider all humankind as a common ingroup.

We hypothesized that people who used artificial intelligence more frequently would identify with humans more strongly. To test this, we first developed and validated the AI Experience Questionnaire to measure the experience of using artificial intelligence technology and applications (Study 1a). Second, we examined the relationship between AI experience and human identity, exploring the mediating role of AI awareness (Study 1a and 1b). Third, we tested whether AI experience could still predict human identity when controlling for AI threats, international mobility, educational level, age, and gender (Study 2). Finally, we explored how culture moderates the correlation between AI experience and human identity (Study 3).

## 2. Study 1a

### 2.1. Materials and Method

#### 2.1.1. Item Development

To measure individuals’ exposure to artificial intelligence applications and technology, the AI Experience Questionnaire needed to cover a wide range of products and events related to artificial intelligence. Thus, item development was based on three considerations. First, we listed mature technology as well as popular applications on the market. Those applications used for specific areas of expertise (e.g., smart agriculture technology) and those used unknowingly by users (e.g., intelligent security systems) were excluded, as this questionnaire was a mass-oriented, subjective report-based measurement tool.

Second, we interviewed three senior practitioners in the AI industry and three users of smart products. They freely discussed how they used artificial intelligence in their daily lives and at work. Based on the interviews, the experience of using AI could be divided into two categories: (1) AI exposure—the frequency of using artificial intelligence products that require little comprehension and are beginner-friendly; and (2) AI experience—the degree to which a person understands how to use artificial intelligence. That is, an experienced artificial intelligence user, trained or practiced to a certain extent, understands enough about artificial intelligence to use it. It is worth mentioning that people outside the AI industry can also have AI experience. First, the extensive integration of AI into various industries has led many people in other fields to use AI in their work and acquire some AI experience. Second, AI experience is gained not only through work but also through user experience with products that require practice to use (for example, drones).

Third, we reviewed the prior research on computer experience scales and drew on their items and methodologies, because AI was initially a sub-discipline of computer science. Finally, all selected items were evaluated, resulting in 13 initial items in the original version of the questionnaire. There were five entries measuring AI experience and eight entries measuring AI exposure, as seen in the Appendix A. All items were scored on a 7-point Likert scale (1 = never used, 7 = frequently used).

#### 2.1.2. Sample

We recruited Chinese citizens over 18 years old and fluent in Chinese through Credamo, using a web-based survey. As Hauser and Schwarz confirmed, when participants are recruited by a web-based survey system, the quality of their responses is not inferior to that of campus participants [36]. As the sample size increases, the sampling error decreases, the sample factor analysis solution becomes more stable, and the true population structure is reflected more accurately [37]. With low communalities and a small number of factors, at least 300 samples are needed [38]. Thus, a final sample of 806 participants (*M*_age_ = 26.94, *SD* = 5.74; 429 males, 377 females; 245 majoring in AI-related fields, 561 majoring in non-AI-related fields) were randomly divided into two groups. Exploratory factor analysis was carried out on the first sample (*n* = 421), and confirmatory factor analysis was carried out on the second sample (*n* = 385).

#### 2.1.3. Measures

Human identity was measured using the Identification With All Humanity (IWAH) scale [11]. Nine items were used for the collection of responses on a 5-point Likert scale. The original version of IWAH was independently translated into Chinese by two PhD candidates in social psychology, and then compared and modified by two other PhD students until a consensus was reached. Because there is no concept of “community” in Chinese culture, only identification with “Chinese” and identification with humankind were rated in the present study. The two scores of human identity and national identity were subtracted to yield the partial score as the final score. The partial score had two advantages: first, it subtracted the overlap of the two identities [11], and second, it could reflect the salience of the human group relative to the subgroup. The higher the final scores, the greater the human identity. The Cronbach’s α was 0.93, indicating that the internal consistency reliability was sufficient.

AI awareness was measured using the sum of the standard scores for perceived anthropomorphism and perceived proximity. First, the perceived anthropomorphism of artificial intelligence was measured by four items. Three of these were adapted from the GODSPEED questionnaire—“machinelike/humanlike; unconscious/conscious; and artificial/lifelike”—and were 7-point Likert scales [22]. The other two items in the GODSPEED questionnaire measure anthropomorphism in appearance, and they were excluded. The last item was “How much do you think the current state of artificial intelligence is human-like?” (1 = very dissimilar, 7 = very similar). The consistency reliability of all four items was 0.86.

Then, the measurement instrument for perceived proximity was adapted from Müller and Bostrom’s survey [19] and consisted of three questions: (1) “How likely do you think it is to achieve human-level artificial intelligence, which can carry out most professions at least as well as a typical human?” (1 = never ever, 7 = totally possible); (2) “To what extent do you think the current state of artificial intelligence has human-level intelligence, which can carry out most professions at least as well as a typical human?” (1 = very distant, 7 = very close); (3) “In your opinion, how many years would it take to achieve human-level artificial intelligence that can carry out most professions at least as well as a typical human?” (1 = very distant, 7 = very close). We determined a Cronbach’s α value of 0.76, which was above the suggested 0.7 threshold and hence indicated satisfactory internal consistency reliability.

### 2.2. Results

#### 2.2.1. Item Analysis

The samples were ranked according to their total scores, with the first 27% (*n* = 83) designated as the high group and the next 27% (*n* = 81) as the low group. An independent two-sample *t*-test was conducted on the scores of the two groups of samples for each item. The findings revealed a significant difference in scores between groups for all items (*p* < 0.001), indicating good discrimination.

#### 2.2.2. Factor Analysis

An exploratory factor analysis was conducted with the first sub-sample (*n* = 421) obtained by randomly splitting the sample in half. The results of Bartlett’s test of sphericity (χ^2^(78) = 2395.71, *p* < 0.001) and the Kaiser–Meyer–Olkin measure of sampling adequacy (KMO = 0.89) indicated that the data were suitable for exploratory factor analysis. Using principal component analysis (PCA) and varimax rotation, two factors were extracted with an eigenvalue greater than one and a 55.79% cumulative percentage of variance. Four items with cross-loading were deleted, and the remaining nine items were subjected to exploratory factor analysis again. The two factors were initially identified as AI experience and AI exposure, explaining 59.26% of the variance, and the factor loadings are detailed in Table 1.

The confirmatory factor analysis was performed with the second sub-sample (*n* = 385). To assess the model fit, we used the following four common fit indices: χ^2^/*df* = 3.70, RMSEA = 0.08, CFI = 0.96, and TLI = 0.94. The two-factor structure was confirmed, showing a very good fit to the data.

#### 2.2.3. Reliability Analysis

The internal consistency coefficients of the AI Experience Questionnaire, AI experience dimension, and AI exposure dimension were 0.84, 0.89, and 0.71, respectively.

#### 2.2.4. Validity Analysis

Structural validity. The confirmatory factor analysis suggested that the structure of this questionnaire was sound. Five items in the AI experience dimension were significantly correlated with the AI experience dimension scores, with correlation coefficients ranging from 0.78 to 0.89. These five items were significantly correlated with each other, presenting correlation coefficients ranging from 0.47 to 0.80. Four items in the AI exposure dimension were significantly correlated with the AI exposure dimension scores, with correlation coefficients ranging from 0.69 to 0.75. These four items were significantly correlated with each other, presenting correlation coefficients ranging from 0.31 to 0.43. The correlation coefficient between each item and the dimension to which it belonged was greater than the correlation coefficient between each item—these items had a strong attribution.

Criterion validity. The results showed that participants majoring in artificial intelligence scored significantly higher than those majoring in non-AI-related disciplines for the AI Experience Questionnaire, the AI experience dimension, and the AI exposure dimension, indicating that the questionnaire measured the intended factors, as seen in Table 2.

#### 2.2.5. Correlation Analysis

What was the relationship between AI experience and human identity? According to a correlation analysis (see Table 3), the AI experience dimension scores were significantly and positively correlated with human identity (*r* = 0.30, *p* < 0.001). The results supported hypothesis 1—AI experience positively predicts human identity.

#### 2.2.6. Mediation Analysis

All variables were significantly correlated with each other. Thus, in case of multicollinearity causing instability in the results, all continuous variables in the equation were standardized, and collinearity was diagnosed. The results showed that the eigenvalues (0.55, 1.45) of all predictor variables were greater than 0, the condition indices (1.00, 1.62) were less than 10, the VIFs (1.25) were less than 5, and the tolerances (0.80) were greater than 0.1—the data did not present severe multicollinearity and could be further analyzed. The regression analysis showed that AI experience significantly and positively predicted AI awareness (β = 0.82, *t*(804) = 14.22, *p* < 0.001) and human identity (β = 0.27, *t*(804) = 7.14, *p* < 0.001), and AI awareness significantly and positively predicted human identity (β = 0.04, *t*(803) = 2.01, *p* = 0.045), as shown in Table 4.

**Figure 1:**
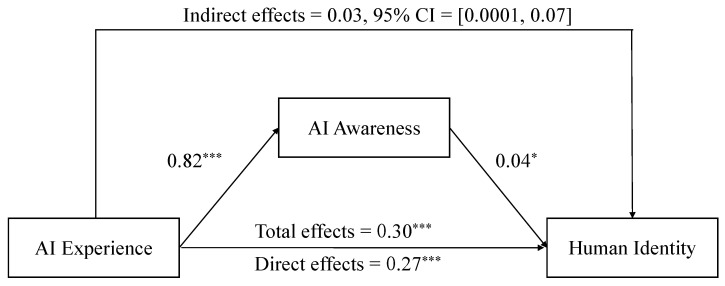
Mediating effect of artificial intelligence awareness. Note: * *p* < 0.05, *** *p* < 0.001.

PROCESS macro in SPSS (Model 4) was utilized to test the mediating effect using the bootstrap method with 5000 replicate samples at 95% confidence intervals [39]. The indirect effect of AI experience on human identity via AI awareness was significant, 95% CI = [0.0001, 0.07], with an effect size of 0.03. When controlling the effect of AI awareness, the direct effect of AI experience on human identity was significant, 95% CI = [0.19, 0.34], with an effect size of 0.27. The results indicated that both the direct effect of AI experience and the mediating effect of AI awareness attained significant levels, which supported Hypothesis 1 and 2, as shown in Figure 1.

### 2.3. Discussion

Study 1a was carried out in the following steps to ensure good psychometric properties: (1) The initial items of the questionnaire were determined by identifying the status of AI applications through consulting the literature and interviews. (2) Item analysis, exploratory factor analysis, confirmatory factor analysis, reliability analysis, and validity analysis were conducted. Finally, the AI Experience Questionnaire contained five items for AI experience and four items for AI exposure. All items were positively rated on a 7-point Likert scale (1 = never used, 7 = frequently used).

A correlation analysis revealed that the total scores of the AI Experience Questionnaire and the AI experience dimension scores positively and significantly predicted human identity, while the correlation between AI exposure and human identity was not statistically significant. In addition, AI experience was associated with AI awareness and human identity more strongly than AI exposure. One possible explanation for the AI experience–AI exposure discrepancy was that an understanding of AI mechanisms may help promote AI awareness and thus enhance human identity, because AI experience requires an understanding of how AI works, whereas AI exposure does not.

The mediation analysis revealed that AI awareness, comprised of perceived anthropomorphism and perceived proximity, partially mediated the relationship between AI experience and human identity. It is important to note that even though AI experience significantly predicted human identity, its correlation coefficient was small. This was because numerous factors influence human identity, and other factors may carry greater weight than the influence of artificial intelligence. This may also be why, in our daily lives, people rarely notice that artificial intelligence is changing the way they see themselves.

## 3. Study 1b

We replicated Study 1a half a year later to test the robustness of the mediation model, using the same measurement instruments and statistical methods.

### 3.1. Method

#### Sample

Chinese citizens over 18 years old were recruited on Credamo. Those who failed the attention check were excluded from further analyses. Final analyses included 886 participants (*M*_age_ = 27.47, *SD* = 6.65; 389 males, 485 females; 244 majoring in AI, 632 majoring in non-AI-related fields).

### 3.2. Results

First, all continuous variables in the equation were standardized (Z-scores), and collinearity was diagnosed. The results indicated that all predictor variables had eigenvalues (0.30, 2.11) greater than 0, conditional indices (1.00, 2.64) less than 10, VIFs (1.39, 1.97) less than 5, and tolerances (0.51, 0.72) greater than 0.1, so the data did not have severe multicollinearity and were suitable for testing the mediating effect.

**Figure 2:**
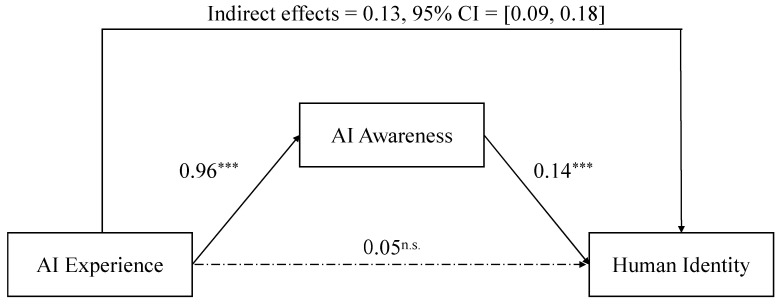
Mediating effect of artificial intelligence awareness. Note: *** *p* < 0.001; n.s. indicates not significant.

PROCESS macro in SPSS (Model 4) was utilized to test the mediating effect using the bootstrap method with 5000 replicate samples at 95% confidence intervals [39]. The mediating effect of AI awareness did not reach 0, 95% CI = [0.09, 0.18], with an effect size of 0.13. When controlling the effect of AI awareness, the direct effect of AI experience on human identity was not significant, 95% CI = [−0.02, 0.12], with an effect size of 0.05, as shown in Figure 2.

### 3.3. Discussion

The results were generally consistent with the results of Study 1a (6 months earlier): AI experience significantly and positively predicted human identity; AI awareness, consisting of AI anthropomorphic perception and human horizontal proximity perception, mediated the relationship between AI experience and human identity. However, unlike Study 1a, the direct effect of AI experience on human identity was not statistically significant in Study 1b, making it difficult to determine the ratio of this direct effect to the total effect of AI experience on human identity. This may have been because the two studies had different samples. Although independent two-sample *t* tests revealed no statistically significant difference between the two samples in the total scores for the AI Experience Questionnaire (*M*_2021.01_ = 42.79, *SD* = 9.62 vs. *M*_2020.06_ = 42.27, *SD* = 10.05, *t*(1690) = 1.09, *p* = 0.28) and for AI experience (*M*_2021.01_ = 19.74, *SD* = 7.51 vs. *M*_2020.06_ = 20.33, *SD* = 7.63, *t*(1690) = –1.59, *p* = 0.11), the first sample had significantly higher AI exposure than the second (*M*_2021.01_ = 23.05, *SD* = 3.58 vs. *M*_2020.06_ = 21.94, *SD* = 3.80, *t*(1690) = 6.17, *p* < 0.001). However, it is worth noting here that both Study 1a and Study 1b demonstrated a significant indirect correlation between AI experience and human identity. This suggests that the indirect effect of AI experience on human identity may be more robust and therefore more important than its direct effects. Taken together, we confirmed the positive correlation between AI experience and human identity through the questionnaires, but the correlation was weak. Therefore, it would be ideal if experimental evidence could be found for future research to justify the effect of AI experience and AI awareness on human identity.

## 4. Study 2

Confounding variables were explored. First, the threat of robots replacing human workers reduces intergroup bias within humans [5,6]. Second, international mobility influences the development of identity [40], as contact can enhance the identification of members of different subgroups with a common ingroup [41]. Third, educational level positively predicts human identity [10]. Fourth, educational level, age, and gender may be correlated with AI experience. Considering that studies on computer experience have demonstrated a significant correlation between computer experience and educational level, age, and gender [42,43], we aimed to test whether AI experience could still significantly and positively predict human identity when controlling for AI threats, international mobility, educational level, age, and gender.

### 4.1. Method

#### 4.1.1. Sample

One thousand and fifty-eight Chinese people over the age of 18 participated in this study through a web-based survey on Credamo. G*Power 3.1 was used to determine the sample size [44]. In G*Power, the linear multiple regression model, fixed model, and *R*^2^ increase options were selected for the *F*-test. The estimate of the effect size *f*^2^ of 0.02 was conservative. To provide 85% power, the a priori power analysis suggested a sample size of 975. Overall, we analyzed data from 981 participants with a mean age of 27.55 ± 6.74 years; 448 males and 533 females; and 273 majoring in AI.

#### 4.1.2. Design and Procedure

After reading the instructions and agreeing to participate in the study, participants sequentially completed the AI Experience Questionnaire and measurement instruments for perceived anthropomorphism, perceived proximity, AI threats, and human identity. Then, their international mobility experience, highest education level, age, gender, and city of residence were recorded.

#### 4.1.3. Measures

All variables were treated as numerical variables based on their attribute values. The same instruments as in Study 1a and 1b were utilized to measure human identity (IWAH, α = 0.93); AI experience (α = 0.81); and AI awareness (the sum of standardized scores for AI anthropomorphic perception (α = 0.87) and human-level AI proximity perception (α = 0.77)). The threat of artificial intelligence was measured by responses to the statement “Artificial intelligence will replace almost all of our jobs” on a scale of 1 to 7 (1 = strongly disagree, 7 = strongly agree). International mobility experience was included and treated as a dichotomous variable. Education level was divided into four levels (below bachelor’s, bachelor’s, master’s, and doctoral) and formed four dummy variables. In addition, AI exposure, age, gender, and residence were also included. The operational definitions of each variable are detailed in Table 5.

### 4.2. Results

Could AI experience still predict human identity when controlling for AI threats, international mobility, educational level, age, and gender? The stepwise regression analysis included two steps: step 1 comprised control variables—AI threats, international mobility experience, educational level, AI exposure, age, gender, and the city of residence; step 2 comprised AI experience; and block 3 comprised AI awareness. The continuous variables in the models were standardized (Z-scores), and collinearity was diagnosed. All the predictors in the three models exhibited eigenvalues (0.08, 3.63) greater than zero, conditional indices (1.00, 6.84) less than ten, VIFs (1.04, 1.82) less than five, and tolerances (0.55, 0.97) greater than 0.1; thus, there was no serious multicollinearity.

In model 2, all predictors accounted for 13.6% of the variance in human identity (*F*(12, 968) = 13.86, *p* < 0.001); AI experience and AI awareness significantly improved the predictive power (Δ*R*^2^ = 0.06, *F*(2, 968) = 35.40, *p* < 0.001). As shown in Table 6, the results showed that AI experience positively predicted human identity when controlling for AI threats, international mobility experience, educational level, AI exposure, age, and gender—supporting Hypothesis 2.

In addition, the regression coefficients for AI threat, AI experience, and AI awareness were significantly positive, while those for AI exposure and age were significantly negative. The highest regression coefficient was for AI awareness (β = 0.21 *t*(968) = 5.56, *p* < 0.001), followed by AI threats (β = 0.14, *t*(968) = 4.35, *p* < 0.001) and AI experience (β = 0.14, *t*(968) = 3.37, *p* = 0.001). The results showed that AI threat, AI experience, and AI awareness had positive and relatively strong correlations with human identity; however, AI exposure and age had a negative and weaker correlation with human identity.

### 4.3. Discussion

Study 2 verified that AI experience positively predicted human identity when controlling for AI threat, international mobility experience, education level, AI exposure, age, and gender. This finding suggests that AI experience may increase the likelihood that people perceive themselves as members of the human race, even in the absence of conflict or threat between humans and artificial intelligence. However, mere exposure to AI is not sufficient to promote human identity, because the association between AI experience and human identity was clearly positive, while AI exposure did not positively predict human identity. This may have been because a person who is only exposed to AI without understanding what makes a machine intelligent, what the nature of artificial intelligence is, and how intelligence can exist independently of life may not perceive artificial intelligence as an outgroup of humans. In short, the social categorization of humans and AI may be the minimum condition for promoting human identity, consistent with corresponding findings in humans alone [45].

The questionnaire study had two major limitations: first, the predictive power of the regression model was weak. One possible explanation was that due to the numerous factors influencing human identity, the mechanism of influence exceeded the predictive power of the regression model. Additionally, the causal relationship between AI experience and human identity needs further validation. The second limitation was the measurement of international mobility. This study revealed that the effect of international mobility experience on human identity did not reach a statistically significant level, contrary to the findings of previous research. One possible explanation is that the effect of transnational mobility experience on common ingroup identity is immediate and of short duration, whereas this study measured a dichotomous variable of international mobility experience over a period of time. Another possible explanation can be found in the contact hypothesis: contact can reduce bias by encouraging more positive perceptions of the outgroup when certain conditions are met. These conditions include equal status between the members, cooperative interdependence, opportunities for personal acquaintance between outgroup members, and egalitarian norms [46]. However, the measurement of international mobility experience in this study was superficial, as only the existence or lack of experience was measured, without discussing or qualifying the above conditions. This may have contributed to the insignificant correlation between international mobility and human identity in Study 2.

## 5. Study 3

Study 3 aimed to explore how culture interacts with AI experience in terms of relevance to human identity and to test the Eastern-amplified hypothesis and the category-based hypothesis.

### 5.1. Method

#### 5.1.1. Sample

The a priori power analysis in G*Power demonstrated that the sample size was 182, with α = 0.05, power = 85%, and effect size *d* = 0.4. We recruited participants born and residing in China through Credamo (*n* = 92) and participants born and residing in the United States through Amazon’s MTurk (*n* = 90). Data from four participants who failed attention checks and two Asian Americans were excluded from further analysis. Final analyses included 177 participants (90 Chinese; *M*_age_ = 32.35 ± 10.99; 103 males).

#### 5.1.2. Procedure

After providing informed consent to take part in the study, participants were asked to fill out the AI Experience Questionnaire, Identification With All Humans, and Self-Construal Scale in turn. Subsequently, their age, gender, and occupation were also collected.

#### 5.1.3. Measures

To assess AI experience, the AI Experience Questionnaire was used, which included the AI experience dimension (α = 0.90) and the AI exposure dimension (α = 0.77). Identification With All Humanity (IWAH; α = 0.92) was also used to measure human identity. However, Study 3 used raw scores of human identity; Studies 1a and 1b used partial scores. That is to say, the human identity measured in Study 3 did not subtract the fraction overlapping with national identity, because national identity may have changed during the COVID-19 pandemic. According to our unpublished findings, Chinese people’s national identity scores were stronger after the outbreak (*M*_after_ = 41.41, *SD* = 4.66; on June 14, 2020; *n* = 182) than before the outbreak (*M*_before_ = 37.91, *SD* = 6.95; on January 8, 2020; *n* = 137; *t*(224) = 5.08, *p* < 0.001, Cohen’s *d* = 0.61); however, the raw scores of human identity were not statistically significantly different (*p* = 0.28). As a result, the partial scores of human identity, obtained by subtracting the national identity scores from the raw scores, significantly decreased (*t*(263) = 3.40, *p* = 0.001, Cohen’s *d* = 0.37). This suggested that the COVID-19 pandemic was significantly more predictive of national identity than human identity. To control confounding variables from the pandemic, we selected raw scores to measure human identity in this cross-cultural study.

### 5.2. Results

Independent two-sample *t* tests for AI exposure, AI experience, and human identity showed that Easterners had significantly higher total scores in the AI Experience Questionnaire (*M* = 45.67, *SD* = 9.08) than Westerners (*M* = 41.00, *SD* = 13.13, *t*(152) = 2.74, *p* = 0.007, Cohen’s *d* = 0.41); Easterners had significantly higher AI exposure (*M* = 23.07, *SD* = 3.16) than Westerners (*M* = 19.76, *SD* = 5.26, *t*(140) = 5.06, *p* < 0.001, Cohen’s *d* = 0.76); but the group difference in AI experience (*p* = 0.26) and human identity (*p* = 0.16) was not significant (see Table 7). The results demonstrated that Easterners were more frequently exposed to artificial intelligence than Westerners, but there was no evidence of a cultural difference in AI experience or human identity.

Did AI experience positively predict human identity in both cultures? We conducted a stepwise regression analysis to control for culture in Step 2. Because the mean age of Eastern participants was lower than that of the Western participants, and *t*-test analysis found that the Easterners had higher scores for AI exposure than the Westerners, we controlled for age and AI exposure in Step 3. All the continuous variables in the regression models were standardized (Z-scores), and collinearity was diagnosed. The results showed that all the predictor variables had eigenvalues (0.16–1.93) greater than 0, conditional indices (1.00–3.46) less than 10, VIFs (1.68–1.73) less than 5, and tolerances (0.51–0.60) greater than 0.1, so the data did not have severe multicollinearity. As shown in Table 8, the regression coefficients for AI experience were significantly positive (β = 0.55 *t*(177) = 8.77, *p* < 0.001) after incorporating culture into Model 2, and they were also significantly positive (β = 0.33 *t*(177) = 4.23, *p* < 0.001) after incorporating culture, age, and AI exposure into Model 3. Furthermore, Model 3 accounted for 38.9% of the variance in human identity (*F*(4, 172) = 28.97, *p* < 0.001). The results suggested that AI experience significantly and positively predicted human identity when controlling for culture, age, and AI exposure.

Was the correlation between AI experience and human identity stronger in Eastern culture or Western culture? Pearson’s correlation analysis showed a weak positive correlation between Easterners’ AI experience and human identity (*r* = 0.36, *p* < 0.001), but a strong positive correlation between Westerners’ AI experience and human identity (*r* = 0.71, *p* < 0.001). The results revealed that AI experience was a significant predictor of human identity in both cultures; however, Westerners’ AI experience was more predictive of human identity than that of Easterners, supporting the category-based hypothesis.

The moderating effect of culture on the relationship between AI experience and human identity was examined. First, we observed a significant interaction between AI experience and culture (β = 0.26, *t*(172) = 2.04, *p* = 0.04), as shown in Table 9.

**Figure 3:**
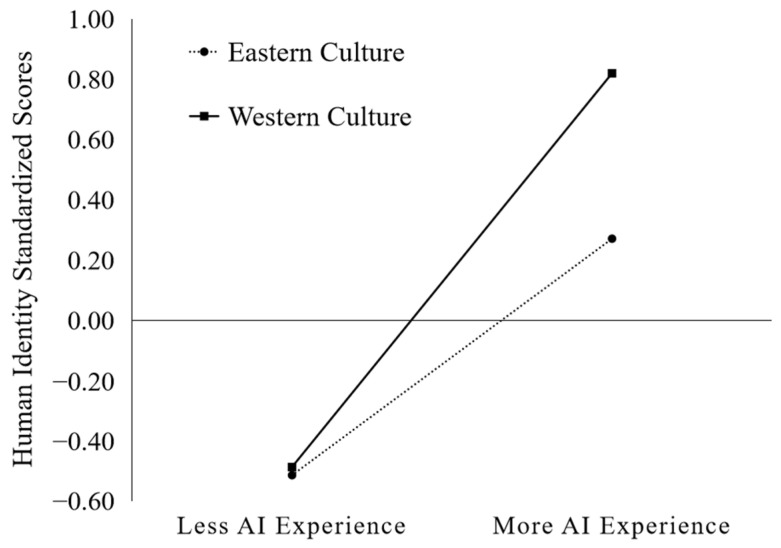
Moderating role of culture in the AI experience–human identity relationship.

PROCESS macro in SPSS (Model 1), using the bootstrap method with 5000 replicate samples at 95% confidence intervals [39], demonstrated that culture significantly moderated the relationship between AI experience and human identity (*R*^2^ = 0.02, *F*(1, 173) = 4.15, *p* = 0.04). As shown in Figure 3, the correlation between AI experience and human identity was stronger in Western culture than in Eastern culture (β = 0.26, *p* = 0.04, 95% CI = [0.01, 0.51]). These moderation analysis results also supported the category-based hypothesis.

### 5.3. Discussion

The results of Study 3 confirmed that both Easterners’ and Westerners’ AI experience had a significant positive correlation with human identity. It is worth noting that there were two cultural differences identified in this study. First, Western culture amplified the correlation between AI experience and human identity compared to Eastern culture. The results of Study 3 supported the category-based hypothesis. Second, the findings also revealed that Easterners had significantly more AI exposure than Westerners. This phenomenon could be explained at both the demand and supply levels: (1) Regarding consumers, Westerners are more concerned about privacy risks and are less connected to AI [29,33], leading to less exposure. (2) Regarding producers, this result may suggest that China is a trend-setter in the development of AI applications, based on self-reported trends.

The primary limitation of Study 3 was the sampling differences between the two cultures. Because the two groups were recruited using two different online questionnaire platforms, the mean age of the Western participants (*M* = 38.87 ± 11.70) was higher than that of the Eastern participants (*M* = 26.03 ± 4.95). The effect of age was statistically controlled for using stepwise regression analysis, confirming the positive correlation between AI experience and human identity in both Eastern and Western culture. However, the moderating role of culture needs further validation, as the moderation analysis did not exclude the confounding effect of demographic variables such as age.

## 6. Discussion

This study explored the relationship between AI experience and human identity as well as the role of AI awareness as a mediator. Both Study 1a and Study 1b confirmed that AI experience significantly and positively predicted human identity. Additionally, this relationship was mediated by AI awareness, which comprised perceived anthropomorphism and perceived proximity. Study 2 was conducted to control for confounding variables. It was confirmed that, when controlling for AI threat, international mobility experience, educational level, AI exposure, age, and gender, AI experience significantly and positively predicted human identity, but AI exposure demonstrated a significant negative association with human identity. Finally, a cross-cultural study confirmed that AI experience had a positive and significant correlation with human identity for both Easterners and Westerners. However, the correlation coefficient between AI experience and human identity in Western culture (*r* = 0.71, *p* < 0.001) was stronger than that in Eastern culture (*r* = 0.36, *p* < 0.001). A moderation analysis demonstrated that Eastern culture amplified the correlation between AI experience and human identity, which supported the category-based hypothesis. These results suggested that persons with more AI experience are more cognitively ready to perceive AI as an outgroup of humans and therefore may identify more strongly with humankind.

### 6.1. Contributions

The findings of this study enrich the factors considered in the common ingroup identity model. Previously, factors such as severe natural disasters [47] or international mobility experience [41] were empirically confirmed. It is now confirmed that AI can also serve as a factor to reduce intergroup bias in humans and enhance human identity. Our findings broadened the social identity approach to include artificial intelligence—a non-human entity—and revealed an unprecedented contextual pattern for self-concept, as different group identifications imply different levels of self-categorization. Concisely, previous studies have found that micro-group identification is stronger than group identification in general [48]. However, our study found that the greater a person’s AI experience, the higher his/her partial score for human identity minus national identity. This implied that acquiring knowledge through exposure to AI strengthened the salience of human identity compared to micro-group identity and could thus truly enhance the relative strength of human identity.

Additionally, the category-based hypothesis was supported by Study 3, suggesting that Westerners are more likely to identify with a common ingroup due to exposure to their respective outgroup. These findings fill a small gap in the study of social identity. As the social identity processes identified from the study of human intergroup relations also apply to human–AI relations, our findings can be generalized to a wider range of intergroup relationships.

Moreover, investigating the social psychological effects of AI experience opens up a range of topics that extend beyond scenarios of direct human–computer interaction and provides a new perspective from which to explore the social impact of AI. No questionnaire measuring AI experience has previously been developed, but the present study filled this gap. The AI Experience Questionnaire can not only be used to measure AI experience and quantitatively analyze the psychosocial effects of AI experience, but it can also be widely applied in other AI social psychology studies—for instance, to test for group differences in AI experience when this is set as a control variable and to screen for specific levels of AI experience when recruiting participants. However, due to the rapid evolution of AI, the AI Experience Questionnaire must be modified to account for the latest research and technological developments, and the specific entries must be iteratively updated to reflect the current performance of artificial intelligence.

These findings also have practical implications for helping people feel more connected to humanity. Historically, social categorization among groups has led people to favor their ingroups [49] and act derogatorily toward outgroups [50]. AI experience may become a new factor in the common ingroup identity model, because the experience of using artificial intelligence technologies and applications enhances their perceived anthropomorphism and perceived proximity, making people inclined to perceive AI as an outgroup object, thus affecting their self-categorization.

### 6.2. Limitations and Future Directions

This study had limitations that could be overcome by certain avenues of future investigation. First, the psychological mechanisms behind different cultures need to be further tested. One explanation is that Westerners’ ingroup identification is dominated by social categories, whereas Easterners’ ingroup identity is formed on the basis of relations, as mentioned in the introduction [34]. Westerners are therefore more sensitive to the categorical distinctions between ingroups and outgroups. An alternative explanation for this observation is that multicultural experiences predict less prejudice via stronger identification with all humanity (IWAH) [51]. The participants of Western culture recruited in this study were from the United States, a multiracial immigrant country. Thus, they had more contact with members of different cultures and experiences with different cultural elements, because they lived in more culturally diverse environments. Future research can investigate whether multicultural experiences are the psychological mechanism behind the moderating role of culture in a manipulative experiment. Furthermore, future research could also manipulate AI threat to the common interests of humans to test the alternative hypothesis and then measure whether Western culture (where ingroup identifications are category-based) still has an amplifying effect. If there are more than categorical cues, Western culture would no longer be shown to have an amplifying effect on human identity. The category-based hypothesis would then be further supported. This would also provide stronger evidence for the change in AI’s social categorization, which is the minimum condition for AI to enhance human identity.

Furthermore, although our conclusions were drawn specifically in the context of comparisons between Western cultures (particularly the United States) and East Asian cultures (particularly China), we believe that the correlation between AI experience and human identity provides a useful framework for viewing cultural effects on social identity processes more broadly. Future studies, conducted in a greater number of countries, could explore these issues further. In addition, the interaction between these factors should be further explored. The moderating role of culture suggests that AI experience acts in combination with other factors, instead of acting independently, so we are also interested in how the effect of contamination varies along with other factors.

## 7. Conclusions

To conclude, the present study examined AI experience as a predictor of human identity. As expected, AI experience positively predicted human identity, even when controlling for AI threat, educational level, international mobility experience, gender, age, and culture. However, mere AI exposure was not significantly positively correlated with human identity. We demonstrate that AI awareness, consisting of perceived anthropomorphism and perceived proximity, mediated the relationship between AI experience and human identity. In addition, for both Easterners and Westerners, the correlation between AI experience and human identity was significantly positive; however, the correlation between Westerners’ AI experiences and human identity was stronger than that of Easterners. Extending previous social identity research, this study implies that AI experience is a positive predictor of the tendency to perceive AI as an outgroup and thus positively correlates with identification with humankind.

## Figures and Tables

**Table 1:** Factor loadings of the AI Experience Questionnaire, Study 1a.

Item No.	*M*(*SD*)	Factor 1	Factor 2
1	3.95 (1.75)	0.88	0.07
2	4.47 (1.79)	0.75	0.24
3	3.55 (1.95)	0.88	0.07
4	3.98 (1.80)	0.77	0.14
5	4.13 (1.82)	0.81	0.19
7	5.63 (1.24)	0.20	0.71
8	5.67 (1.21)	0.08	0.75
10	5.77 (1.23)	0.07	0.69
11	5.40 (1.38)	0.15	0.72

**Table 2:** AI Experience Questionnaire scores for AI majors and non-AI-related majors, Study 1a.

	AI Majors	Non-AI-Related Majors	*t*	Cohen’s *d*
Age	27.24 ± 5.59	26.80 ± 5.80	1.00	0.32
Total Score	47.77 ± 7.38	40.52 ± 9.77	11.57 ***	0.84
AI Experience	24.70 ± 5.65	18.42 ± 7.59	13.01 ***	0.94
AI Exposure	23.07 ± 3.29	22.11 ± 3.96	3.61 ***	0.26
*n*	245	561		

Note: *** *p* < 0.001.

**Table 3:** Mean (*M*) and standard deviation (*SD*) of each variable and its Pearson correlation matrix, Study 1a.

		*M*	*SD*	1	2	3
1	Human Identity	−12.84	8.11	—		
2	Perceived Anthropomorphism	18.94	5.25	0.20 ***	—	
3	Perceived Proximity	15.30	3.64	0.16 ***	0.67 ***	—
4	AI Experience	20.33	7.63	0.30 ***	0.41 ***	0.41 ***
5	AI Exposure	22.40	3.79	0.06	0.16 ***	0.17 ***
6	Total Score	42.73	9.70	0.26 ***	0.39 ***	0.38 ***

Note: *** *p* < 0.001.

**Table 4:** Regression analysis of variables in the model, Study 1a.

		Overall Fit Index			Significance of Coefficients	
Outcome Variable	Predictors	*R*	*R* ^2^	*F*	β	*t*
AI Awareness	AI Experience	0.45	0.20	202.11 ***	0.82 ***	14.22
Human Identity	AI Experience	0.31	0.10	042.47 ***	0.27 ***	7.14
	AI Awareness				0.04 *	2.01

Note: the variables in the model were standardized; * *p* < 0.05, *** *p* < 0.001.

**Table 5:** Variable selection, Study 2.

	Predictors	Operational Definition	Value
Explanatory Variables	AI Experience	Experience in using AI that requires some understanding and is difficult to operate; the AI experience dimension score of the AI Experience Questionnaire.	5 items, 7-point Likert scale
	AI Awareness	The sum of standardized scores for perceived anthropomorphism and perceived proximity.	7 items, 7-point Likert scale
Control Variables	AI threats	Perceived threat of AI replacing human jobs.	1 item, 7-point Likert scale
	International mobility experience	Whether or not the respondent had experience abroad.	0 or 1
	Education 1	Dummy variable of educational level: whether the highest academic qualification was a bachelor’s degree.	0 or 1
	Education 2	Dummy variable of educational level: whether the highest academic qualification was a master’s degree.	0 or 1
	Education 3	Dummy variable of educational level: whether the highest academic qualification was a PhD degree.	0 or 1
	AI Exposure	Experience in using AI applications that do not require comprehension and are novice friendly; the AI exposure dimension score of the AI Experience Questionnaire.	4 items, 7-point Likert scale
	Age	The number of years the respondent had lived.	Years of age
	Gender	Men = 0, female = 1.	0 or 1
	City 1	Dummy variable of city of residence; whether the respondent lived in one of the four cities belonging to tier one: Beijing, Shanghai, Guangzhou, and Shenzhen.	0 or 1
	City 2	Dummy variable of city of residence; whether or not the respondent lived in a new tier-one city (according to the “City Business Attractiveness Ranking” published by First Finance in 2019)	0 or 1

**Table 6:** Stepwise regression analysis of human identity, Study 2.

	Model 1		Model 2	
Predictor	β	95% CI for *B*	β	95% CI for *B*
Step 1				
AI threat	0.24 ***	[0.18, 0.30]	0.14 ***	[0.08, 0.21]
International mobility experience (yes =1)	0.08 *	[0.03, 0.29]	0.05	[−0.03, 0.23]
Education 1 (bachelor’s = 1)	−0.08	[−0.33, 0.01]	−0.07	[−0.31, 0.02]
Education 2 (master’s = 1)	−0.06	[−0.42, 0.05]	−0.04	[−0.34, 0.11]
Education 3 (PhD = 1)	−0.04	[−1.03, 0.20]	−0.02	[−0.77, 0.42]
AI exposure	0.01	[−0.05, 0.07]	−0.10 **	[−0.16, −0.03]
Age	−0.05	[−0.11, 0.01]	−0.11 ***	[−0.17, −0.05]
Gender (women = 1)	0.13 ***	[0.14, 0.39]	0.14 ***	[0.15, 0.39]
City 1 (tier-one = 1)	−0.02	[−0.20, 0.10]	−0.02	[−0.18, 0.11]
City 2 (new tier-one = 1)	−0.06	[−0.27, 0.02]	−0.05	[−0.24, 0.35]
Step 2				
AI experience	—	—	0.14 **	[0.06, 0.21]
AI awareness	—	—	0.21 ***	[0.07, 0.15]
Constant	−0.003	[−0.17, 0.17]	−0.02	[−0.19, 0.14]
Sample size *n*	981		981	
Adjusted *R*^2^	0.08 ***		14 ***	
Δ*R*^2^			0.06 ***	

Note: CI = confidence interval; the variables in the model were standardized; * *p* < 0.05, ** *p* < 0.01, *** *p* < 0.001.

**Table 7:** *T*-test analysis of AI exposure, AI experience, and human identity, Study 3.

	Eastern Culture		Western Culture			
Variables	*M*	*SD*	*M*	*SD*	Cohen’s *d*	*t*
Total Scores of the AI Experience Questionnaire	45.67	9.08	41.00	13.13	0.41	2.74
AI Experience	22.59	6.97	21.24	8.90	0.17	1.12
AI Exposure	23.08	3.16	19.76	5.27	0.76	5.06 **
Human Identity	30.53	6.96	32.05	7.44	−0.21	−1.40
*n*	90		87			

Note: ** *p* < 0.01.

**Table 8:** Stepwise regression analysis of human identity, Study 3.

	Model 1		Model 2		Model 3	
Predictor	β	95% CI for *B*	β	95% CI for *B*	β	95% CI for *B*
Step 1						
AI Experience	0.54 ***	[0.41, 0.66]	0.55 ***	[0.43, 0.68]	0.33 ***	[0.17, 0.48]
Step 2						
Culture (Western = 1)	—		0.15 *	[0.05, 0.54]	0.35 ***	[0.38, 0.97]
Step 3						
AI Exposure	—				0.39 ***	[0.23, 0.58]
Age	—				−0.13	[−0.26, 0.02]
Constant	0.09		−0.14 *		−0.33 **	
Sample Size *n*	177		177		177	
Adjusted *R*^2^	0.29 ***		0.31 ***		0.39 ***	
Δ*R*^2^			0.02 *		0.09 ***	

Note: CI = confidence interval; the variables in the model were standardized; * *p* < 0.05, ** *p* < 0.01, *** *p* < 0.001.

**Table 9:** Multiple linear regression with interaction, Study 3.

		Fit Index			Significance of Regression Coefficients	
Outcome Variable	Predictor	*R*	*R* ^2^	*F*	β	*t*
Human Identity	AI Experience	0.57	0.33	28.45 ***	0.39 ***	3.92
	Culture				0.29 *	2.35
	AI Experience * Culture				0.26 *	2.04

Note: the variables in the model were standardized; * *p* < 0.05, *** *p* < 0.001.

## Data Availability

The authors will share data from the study upon reasonable request to the corresponding author.

## Appendix A. AI Experience Questionnaire

Please indicate your experience with artificial intelligence. 1 = never, 7 = regularly.

**Dimension**	**Item**
AI experience	1. Using algorithms and methods such as Naïve Bayes, Support Vector Machine, Linear Regression, Logistic Regression, KNN, K-means, Decision Tree, Random Forest, CART, Apriori Machine Learning Algorithm, PCA, CatBoost, ID3, Hierarchical Clustering, Back-Propagation, AdaBoost, Deep Learning, Gradient Boosting Algorithm, Hopfield Network, C4.5, etc.
	2. Using frameworks such as TensorFlow, PyTorch, Keras, etc.
	3. Researching, understanding, and leveraging the following applied technologies: Text Analytics and NLP, Speech Recognition, Virtual Agents, Machine Learning Platforms, Deep Learning Platforms, AI-optimized Hardware, Decision Management, Biometrics, RPA, etc.
	4. Using semi-autonomous driver-assist systems such as active park assist, adaptive cruise control, lane-departure warning, blind-spot monitoring, etc.; or using an unmanned aerial vehicle (UAV).
	5. Using AI-based photo editors or graphic design tools (e.g., AutoDraw and Prisma), or producing hyper-realistic videos using artificial intelligence applications such as Deepfake technology.
AI exposure	1. Using an intelligent virtual assistant (IVA) or intelligent personal assistant (IPA) such as Apple Siri, Microsoft Cortana, Amazon Echo and Alexa, Google Assistant, etc.
	2. Using automatic voice recognition and generation systems (e.g., speech to text, text to speech) through instant messengers, note-taking apps, ebook reader apps, short-form video apps, etc.
	3. Using automatic image detection, recognition, and classification technologies through image search engines, smart photo libraries, online shopping apps, etc.
	4.Using automatic face recognition technology, voice biometric authentication, or a finger vein scanner with respect to security and fraud prevention, access and authentication, biometric payment, etc.
